# A Scoping Review of Nutritional Biomarkers Associated with Food Security

**DOI:** 10.3390/nu15163576

**Published:** 2023-08-14

**Authors:** Lev Krasnovsky, Aidan P. Crowley, Fawaz Naeem, Lucy S. Wang, Gary D. Wu, Ariana M. Chao

**Affiliations:** 1 Perelman School of Medicine, University of Pennsylvania, Philadelphia, PA 19104, USA; aidan.crowley@pennmedicine.upenn.edu (A.P.C.); fawaz.naeem@pennmedicine.upenn.edu (F.N.); lucy.wang@pennmedicine.upenn.edu (L.S.W.); 2 Division of Gastroenterology and Hepatology, Perelman School of Medicine, University of Pennsylvania, Philadelphia, PA 19104, USA; gdwu@pennmedicine.upenn.edu; 3 Johns Hopkins School of Nursing, Johns Hopkins University, Baltimore, MD 21205, USA; ariana.chao@jhu.edu

**Keywords:** metabolomics, nutrition, food security, nutrition security, review, dietary assessment methodologies, biomarkers of nutritional status

## Abstract

Food insecurity affects more than 40 million individuals in the United States and is linked to negative health outcomes due, in part, to poor dietary quality. Despite the emergence of metabolomics as a modality to objectively characterize nutritional biomarkers, it is unclear whether food security is associated with any biomarkers of dietary quality. This scoping review aims to summarize studies that examined associations between nutritional biomarkers and food security, as well as studies that investigated metabolomic differences between people with and without food insecurity. PubMed, Embase, Scopus, and AGRICOLA were searched through August 2022 for studies describing food insecurity and metabolic markers in blood, urine, plasma, hair, or nails. The 78 studies included consisted of targeted assays quantifying lipids, dietary nutrients, heavy metals, and environmental xenobiotics as biochemical features associated with food insecurity. Among those biomarkers which were quantified in at least five studies, none showed a consistent association with food insecurity. Although three biomarkers of dietary quality have been assessed between food-insecure versus food-secure populations, no studies have utilized untargeted metabolomics to characterize patterns of small molecules that distinguish between these two populations. Further studies are needed to characterize the dietary quality profiles of individuals with and without food insecurity.

## 1. Introduction

Food insecurity (FI) is a social determinant of health that affects over 40 million individuals in the United States [1]. Worldwide, the prevalence of moderate to severe food insecurity is approximately 22% [2]. Food insecurity is defined as a lack of reliable access to sufficient quantities of nutritious food and can be classified at an individual or household level into high, marginal, low, and very low food security [1]. It is associated with negative health outcomes such as obesity [3], cardiovascular disease [4], asthma [1], exacerbation of type 2 diabetes [5], and overall mortality [6]. The effects of food insecurity span the spectrum of age, associating with increased hospitalizations in childhood [7] and functional limitations in the elderly [8]. The COVID-19 pandemic has further exposed existing disparities in food security, highlighting how race, income, and mental health characteristics affect vulnerability to FI [9].

For decades, policies have concentrated on combatting food insecurity by ensuring adequate caloric supply [10]. However, many of the present-day adverse health effects of food insecurity are attributed to reduced dietary quality, not quantity [11]. Organizations such as the USDA are beginning to place a new emphasis on nutrition security, distinct from food security, for policy goals aimed at improving population health and wellness [12]. Nutrition security is differentiated from food security by emphasizing consistent, affordable access to foods that promote wellbeing and prevent disease [10]. While the formal definition of FI does acknowledge access to nutritious foods, common public health screening tools are often limited in the amount they screen for this element. For example, the U.S. Department of Agriculture’s (USDA) Household Food Security Survey Module (HFSSM) offers a validated approach for assessing FI risk, but is not designed to assess dietary quality or dietary diversity [13]. References to “balanced meals” are made in this module, but they are not enough to infer nutritional quality [13].

The significant health effects of nutrition insecurity underscore the need for improved evaluation tools and solutions. Comprehensive dietary assessment methods such as food frequency questionnaires or 24-h recalls can provide information on dietary quality, but face tradeoffs regarding the skill and quantity of time they take to administer, as well as possible response bias [13]. This impedes their scalability to population-level scenarios, forcing a compromise between how much information from respondents is collected, the quality of the collected data, and the number of individuals that can be evaluated. Alternate strategies for nutritional status assessment may involve analyzing metabolite profiles in body fluids (i.e., metabolomics) for signatures of dietary patterns. Such analyses have the potential to be both scalable and detailed in assessing nutritional status, and indeed have already been performed on the scale of several thousand individuals [14].

To develop a novel metabolomics-based nutritional screener, reproducible biomarkers associated with dietary exposures must be identified. A number of studies, including those utilizing data from the National Health and Nutrition Examination Survey (NHANES), have found that food insecurity is related to lower intake of fruits and vegetables [15] but not grain, meat, or dairy intake [16]. Likewise, a recent systematic review has identified 69 metabolites representing biomarkers in 11 food- or dietary pattern-specific categories, such as fruit intake, vegetable intake, grain intake, meat intake, and more [17]. Other studies have identified plasma biomarkers associated with the Healthy Eating Index, the Alternate Mediterranean Diet Score, the WHO Healthy Diet Indicator, and the Baltic Sea Diet, as well as dietary biomarkers in urine [18,19]. Despite the known differences in dietary intake between individuals with and without FI, as well as the existence of biomarker candidates representing some of these differences, no studies have systematically reviewed the nutritional metabolite profiles of individuals with and without FI. The aim of this scoping review is to summarize published studies that measure the relationship between food insecurity and nutritional biomarkers, providing a map of existing bioanalytical efforts that may guide future methods in nutrition epidemiology.

## 2. Materials and Methods

### 2.1. Conducting the Search

This scoping review was conducted according to PRISMA-ScR (Preferred Reporting Items for Systematic Reviews and Meta-Analyses for Scoping Reviews) guidelines. The databases PubMed, Embase, Scopus, and AGRICOLA were used for the literature search. The search strategy was created using relevant terms describing food insecurity (and its variants) and biomarkers, including the medium of the biomarker sample. The search terms were refined with the aid of a research librarian to optimize the selection of articles without compromising the sensitivity of the search. Controlled vocabulary supplemented with keywords was utilized to search for studies describing food insecurity and metabolic markers in blood, urine, plasma, hair, or nails as measures of dietary intake. Search terms are listed in supplemental Figure S1. Covidence software was used for screening, extraction, and data management [20]. The search strategy was organized with the following syntax: (“food secur*” OR “food insecur*” OR “food sufficien*” OR “food insufficien*” OR “food access” OR “food desert*” OR “food swamp*”) AND (metabolite* OR metabolom* OR “metabolic syndrome*” OR “diet survey*” OR “diet questionnaire*” OR “diet screener*” OR “dietary nutrient*” OR blood OR urine OR plasma OR hair* OR nail*). The original search was conducted in October 2021, and an update was conducted in August 2022.

### 2.2. Study Selection

Included studies were primary papers or abstracts that assessed participant food security and measured dietary biomarkers; non-primary literature, such as viewpoints, editorials, and systematic reviews, was excluded. The search was limited to articles written in English and Spanish without any restriction on date. The participants in included studies were pediatric or adult populations from any country in whom at least one nutritional biomarker was studied and food security or sufficiency status was documented. The quantification of biomarkers was required to have been stratified by food security category. Comparisons between biomarker levels of food-secure versus -insecure populations were made within individual studies. The outcome assessed between studies was the presence and directionality of association between food security status and a particular biomarker.

### 2.3. Inclusion and Exclusion Criteria

As a prerequisite for inclusion, study populations must not have been exclusively in a particular disease state (e.g., consisting entirely of participants who are HIV-positive). This step was taken because associations identified within populations exclusively with a disease may not be generalizable. Studies that examined people with obesity, as well as populations that had not yet crossed disease thresholds (e.g., populations with prediabetes), were not excluded.

The biomarkers included in the study were any nutrient, mineral, xenobiotic, or food metabolite with known or unknown function. Of note, the following markers were not included: fasting glucose, HbA1C, hemoglobin, markers of inflammation (e.g., D-dimers, C-reactive protein), and markers of oxidative stress (e.g., glutathione). Fasting glucose levels and HbA1c are chronic measures that are not affected by a single meal, and the remaining markers can be associated with dietary patterns but do not reflect specific dietary exposures. Studies that examined biomarkers within a larger diagnosis, such as metabolic syndrome, were excluded if no specific association between the biomarker and food security was reported. Because this review was focused on studies designed to look at associations of biomarkers with food insecurity, studies which only investigated food insecurity as a covariate for a different association were excluded.

### 2.4. Data Extraction and Analysis

Four investigators (A.P.C., L.K., F.N., L.S.W.) screened titles and abstracts of search results. Each abstract was reviewed independently by two reviewers to evaluate eligibility according to established inclusion criteria. Discrepancies were resolved by group discussion and consensus. Full-text screening was conducted independently and in duplicate by A.P.C., L.K., F.N., and L.S.W., and discrepancies were resolved by consensus.

Extracted data included publication year, country, study type, participant description, study setting, number of participants, age, BMI, sex distribution, food insecurity scale, scope of food insecurity scale, food insecurity rate among participants, biomarker collection source, biomarker type, odds ratio of biomarker levels between food security subtypes, significance of relationship, and adjusted covariates. If both adjusted and unadjusted values regarding an association were presented, the adjusted data were used for characterization of relationship direction. For studies which distinguished between different severities of food insecurity (e.g., mild, moderate, severe), significant association between biomarker level and any severity category was recorded as presence of a relationship. Additionally, if only a defined subset of participants demonstrated a significant relationship between food security status and a biomarker, the finding was counted as showing a significant relationship for that population.

Due to heterogeneity between populations, geographies, food security measures, age groups, cultural practices, adjusted covariates, and collection methods, weighted quantitative analysis was not performed. Instead, the directions of relationships between reviewed biomarkers and food insecurity were tabulated for each study and results were tallied into categories showing positive, negative, or non-statistically significant (e.g., *p* > 0.05) association.

## 3. Results

**Figure 1:**
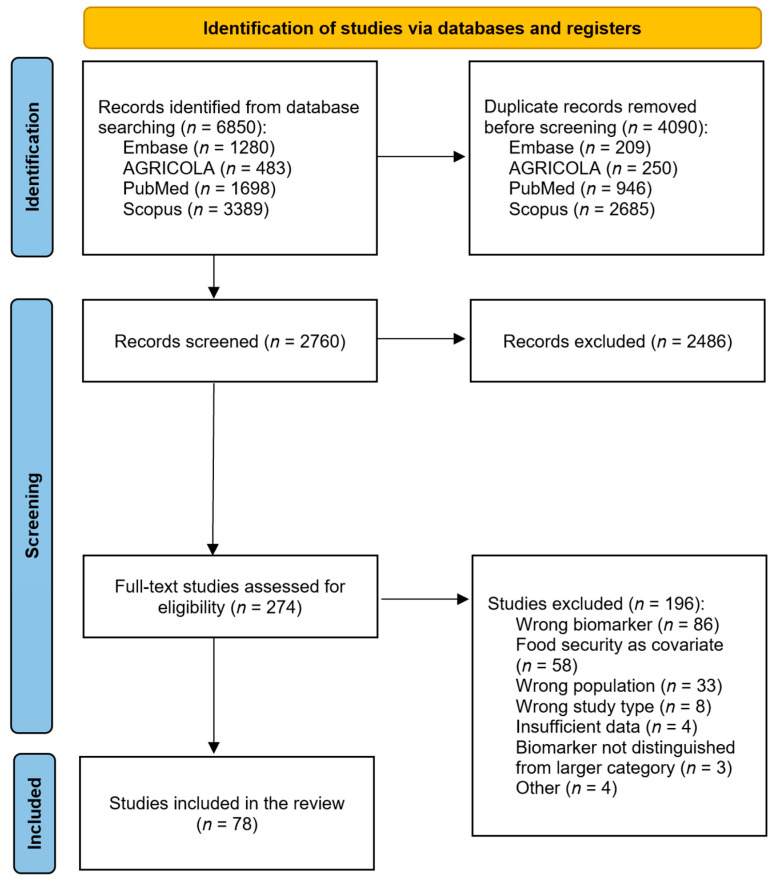
PRISMA flow diagram of the literature search and screening process for a systematic review of nutritional biomarkers associated with food security.

A total of 6850 studies were identified by the search query. Following removal of 4090 duplicates, 2760 studies were screened on the basis of titles and abstracts, leaving 274 for full-text review. Of these, 78 met inclusion criteria and were included in the present analysis. Figure 1 shows the PRISMA diagram for the screening process.

### 3.1. Study Characteristics

The key characteristics of the 78 included studies are summarized in Table A1 [21,22,23,24,25,26,27,28,29,30,31,32,33,34,35,36,37,38,39,40,41,42,43,44,45,46,47,48,49,50,51,52,53,54,55,56,57,58,59,60,61,62,63,64,65,66,67,68,69,70,71,72,73,74,75,76,77,78,79,80,81,82,83,84,85,86,87,88,89,90,91,92,93,94,95,96,97,98]. All included studies were published between 2000 and 2022. Thirty-two studies (41%) were conducted in the US; the remainder were conducted in countries including Malaysia, Iran, Azerbaijan, Colombia, India, Mexico, Bangladesh, Ethiopia, Kenya, Vietnam, Canada, Brazil, Ecuador, Uganda, Ghana, Tanzania, Mongolia, and Pakistan. Two studies (2.6%) used a longitudinal cohort study design, while the remaining seventy-six (97.4%) were cross-sectional. Nineteen studies (24.4%) used NHANES data on food security and serum micronutrient levels. The USDA food security scales, including the HFSSM (which has validated translations and a shortened 6-item version) and the Adult Food Security Survey Module, were used by 35 (44.9%) of the studies. The next most commonly used tool was the Household Food Insecurity Access Scale (HFIAS), which was used by 15 (19.2%) of the studies. Among the pediatric studies, food security was measured at the household level in 25 out of 27 (92.6%) studies, while, among adult studies, household measures were used in 42 out of 47 (89.4%) studies (the remaining studies assessed food security through derivative measures). Nearly all (73 out of 78) of the studies obtained biomarker data solely from blood samples; three used urine samples, and two used both blood and urine. 

### 3.2. Associations between Food Insecurity and Biomarkers

**Figure 2:**
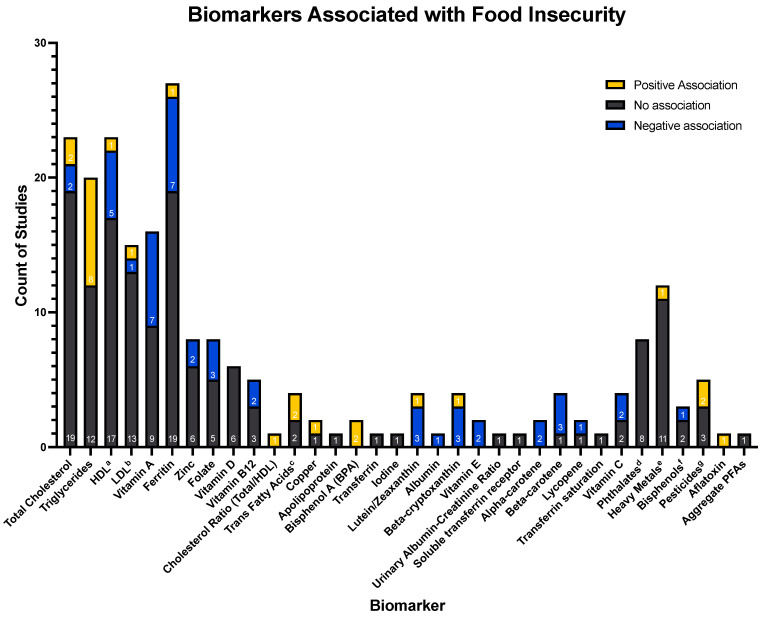
Counts of research articles demonstrating positive, negative, or null associations between levels of a given biomarker and the presence of food insecurity. Certain biomarkers investigated by only one study were grouped together under broader categories, defined below. The sign following each biomarker indicates its individual relationship with food insecurity, with (+) indicating the biomarker is positively associated with the presence of food insecurity, (0) indicating the biomarker is not associated with the presence of food insecurity, and (−) indicating the biomarker is negatively associated with the presence of food insecurity.

Across the 78 included studies, 59 unique biomarkers were analyzed for their association with food insecurity. The total numbers of studies showing statistically significant positive or negative associations, as well as absence of association, are depicted for each biomarker in Figure 2. The ten biomarkers investigated by at least five studies each were total cholesterol, high-density lipoprotein (HDL), low-density lipoprotein (LDL), triglycerides, ferritin, folate, zinc, vitamin A, vitamin B12, and vitamin D. No uniform associations were found between food security and any of these biomarkers. Among the biomarkers evaluated by between two and five studies, vitamin E, alpha-carotene, and bisphenol A (BPA) showed exclusively unidirectional associations across studies (BPA was positively associated with food insecurity, while vitamin E and alpha-carotene were negatively associated). All of the remaining biomarkers were evaluated by either only one study, or did not demonstrate consensus of relationship directionality. Beta-cryptoxanthin, alpha-carotene, and beta-carotene were the only identified biomarkers known to be associated with dietary quality.

Subgroup analyses were performed by geographic location (US vs. non-US) and age-group (non-pregnant adult vs. child under 18). No uniform directional relationships between food insecurity and any biomarker were found for any of these subpopulations (supplemental Figures S2–S6). Similarly, subgroup analysis limited to only NHANES data did not show any new or notably strengthened relationships (supplemental Figure S7).

Key:
^a^ = High-density lipoprotein;
^b^ = Low-density lipoprotein;
^c^ = Associations of trans fatty acids: Trans-9-hexadecenoic acid (0), Trans-11-octadecenoic acid (0), Trans-9-octadecenoic acid (+) and trans-9, Trans-12-octadecenoic acid (+);
^d^ = Associations of phthalates: Mono-2-ethylhexyl phthalate (0), Mono-(2-ethyl-5-oxohexyl) phthalate (0), Mono-(2-ethyl-5-hydroxyhexyl) phthalate (0), Mono-(2-ethyl-5-carboxypentyl) phthalate (0), Mono-n-butyl phthalate (0), Monoisobutyl phthalate (0), Mono-benzyl phthalate (0), Mono-ethyl phthalate (0);
^e^ = Associations of heavy metals: Antimony (+), Barium (0), Beryllium (0), Cadmium (0), Cesium (0), Molybdenum (0), Lead (0), Platinum (0), Thallium (0), Tungsten (0), Uranium (0);
^f^ = Associations of bisphenols: 4-tert-octylphenol (0), Benzophenone-3 (−), Triclosan (0);
^g^ = Associations of pesticides: 2,5-dichlorophenol (0), O-phenyl phenol (0), 2,4-dichlorophenol (0), 2,4,5-trichlorophenol (+), 2,4,6-trichlorophenol (+);
^h^ = Aggregate per- and poly-fluoroalkyl substances, not separated by category (perfluoronanoic acid (PFNA), perfluorooctanesulfonic (PFOS) acid, perfluorooctanoic acid (PFOA), methyl-perfluorooctane sulfonamide acetic acid (Me-PFOSA-AcOH), and perfluorohexanesulphonic acid (PFHxS).

## 4. Discussion

The objective of this scoping review was to summarize the existing literature investigating the association between food security status and physiologic concentrations of nutritional biomarkers. No studies were found that used untargeted metabolomics to investigate this question. Among the studies using targeted analyses, the 10 biomarkers investigated by at least five studies—total cholesterol, HDL, LDL, triglycerides, ferritin, folate, zinc, vitamin A, vitamin D, and vitamin B12—all showed predominantly no relationship with food security status.

Previous reviews have examined the relationships between food insecurity and intake levels of specific nutrients. There is no or very limited evidence suggesting adverse association between food insecurity and intake of vitamin B12, folate, or iron [99]. Likewise, there is very limited evidence of association between food insecurity and total fat intake [99,100]. More evidence exists for adverse association between food insecurity and intakes of vitamin A and zinc [99]. While intake levels serve as an important proxy for serum concentrations, metabolic and environmental differences make it impossible for them to completely reflect physiologic concentrations.

A notable finding of the present review was the relative paucity of studies evaluating patients with and without FI for biomarkers of specific food categories associated with dietary quality; none of the 10 most-identified biomarkers were dietary metabolites. Total cholesterol, LDL, HDL, triglycerides, and ferritin are susceptibility biomarkers, which can measure the impact of a nutritional exposure on host physiology, while folate, iron, vitamin A, vitamin D, and vitamin B12 are micronutrients (non-specific exposure biomarkers) [101]. Only 3 of the 59 identified biomarkers were dietary metabolites associated with specific food group exposures (per the collection identified in [17])—beta-cryptoxanthin, alpha-carotene, and beta-carotene. These showed predominantly negative associations with FI, but were reviewed by fewer than five studies each. Thus, the biomarkers for which there were the most data were biomarkers of general nutritional status, not specific dietary exposure.

The lack of consistent directional relationships between food security status and physiologic concentrations of biomarkers may have had to do with the inherent limitation of using food insecurity as a proxy for nutrition quality. Individuals with food insecurity can be food-insecure in different ways, most notably by over-versus undernutrition. These states would be expected to have different biochemical profiles, and combining them under the umbrella of FI increases the heterogeneity of the population, potentially blurring relationships. Nutrition insecurity, on the other hand, would likely have a more consistent biochemical signature, but, at present, is not as robustly measured in population-level studies as FI. Both measures may nevertheless share explanations for relationships with physiologic concentrations of specific biomarkers. To this aim, a summary of explanations extracted from included studies for the relationships, or lack thereof, between the 10 most-reviewed biomarkers and food security is provided below.

### 4.1. Lipid-Related Markers—Total Cholesterol, HDL, LDL, and Triglycerides

Overall, out of the 78 total studies in this review, the majority of those examining lipid markers (total cholesterol, HDL, LDL, and triglycerides) found no significant association with food insecurity. Generally, researchers cited sample size, non-random sampling, non-fasting serum measurements, and unexpectedly high prevalence of dyslipidemias as explanations for this lack of relationship [52,96]. Nevertheless, the predominant lack of association between food insecurity and lipid markers has also been identified elsewhere, as noted in a recent meta-analysis [102]. Similar to the present findings, the authors of the meta-analysis also concluded that current data do not suggest that there are consistent relationships between dyslipidemias and food insecurity status.

Of the studies that identified significant associations between the presence of dyslipidemias and food insecurity, many cited obesity as a mediating factor. Obesity is known to play a key role in generating many of the hallmarks of dyslipidemia, including low HDL-C. Increased remnants of chylomicrons and very-low-density lipoprotein (VLDL) molecules paired with reduced lipolysis in obesity result in diminished HDL as well as diminished HDL function [103]. Moreover, three out of the eight studies that found a positive association between FI and triglyceride levels were conducted with women [49,77,92]. Studies have reported that women, but not men, who experience food insecurity are more likely to have overweight or obesity [104,105]. Therefore, the high prevalence of hypertriglyceridemia was hypothesized to be mediated by obesity and differences in abdominal fat distribution among these women [73,77,106]. Sex-based differences in hormonal regulation and metabolism of lipids may also explain some of this association [73].

Other explanations for associations between FI and dyslipidemias derive from numerous factors. One hypothesis is centered on individuals with FI demonstrating decreased consumption of antioxidants. Decreased antioxidant defense reduces protection against the peroxidation of HDL, and oxidatively modified HDL demonstrates loss of its antiatherogenic properties [92,107]. Other hypotheses posit that individuals with FI may participate in limited physical activity and experience greater psychosocial stress, which may explain associations with dyslipidemia due to dysregulated cortisol and metabolic hormones [73]. Finally, one author indicated that food-insecure individuals may display patterns of overeating during times of food availability in expectation of future food shortages [49]. If this behavior occurred during the time period of sample collection, it could have explained the lipid marker elevation.

### 4.2. Zinc

Zinc was investigated as a biomarker in eight studies, in which six found no association with food insecurity [57,64,65,67,80,83] and two found a negative association [44,81]. Of the two studies that found an association, one was conducted on Colombian children 1–4 years of age [81] and one was conducted on pregnant women in Ethiopia [44]. Decreased access to zinc-rich foods such as meats, as well as a higher prevalence of plant-based diets rich in zinc-absorption inhibitors such as fiber and phytic acid, were proposed as explanations for why these food-insecure populations were more likely to have lower zinc concentrations. It was also noted that zinc serum concentrations can be altered by contamination when acquiring or processing a sample [81]. This collection technique-associated variance is important to consider in the interpretation of results from large-scale studies. The six studies that found no association studied US children [57], Bangladeshi children living in a slum [65], Ghanian women aged 18–35 [83], Brazilian preschoolers [80], and Colombian children [64,67]. In some of these countries, culturally relevant dietary sources of zinc are generally uncommon, so the whole population may have low zinc levels [80]. This would lower observed differences between food-secure and -insecure populations. Likewise, in populations with broad access to affordable foods rich in zinc, differences in zinc levels would once again be minimized.

### 4.3. Ferritin

A total of 27 studies analyzed the relationship between ferritin concentration and food insecurity, with 7 finding a negative association, 1 finding a positive association, and 19 finding no association [23,24,25,31,34,35,36,42,47,48,53,54,55,56,57,58,63,65,67,72,76,78,79,82,83,85,94]. The studies that identified a negative association attributed this relationship to chronic iron deficiency in the diet [54], parents giving up iron-rich foods to their children and not getting enough iron themselves [54], increased inflammation reducing iron absorption [55], decreased access to heme sources of iron [76], decreased knowledge about iron supplementation [76], shorter inter-pregnancy interval with insufficient time to restore iron stores [48], and increased rates of parasitic infections that may reduce iron absorption [47]. The single study that found a positive association was conducted among Inuit adults who had high consumption of traditional foods, which are often rich in iron [108]. Moreover, the authors noted that traditional food consumption varies throughout the year, so, if the data were collected during a different season, the result may have differed. Among the studies that found no association, explanations for the results included high background of iron deficiency among the population [24,58], effective distribution of iron-rich foods through food assistance programs [25], improved iron absorption from decreased inflammation (achieved through high intakes of traditional foods rich in anti-inflammatory long-chain polyunsaturated fatty acids) [53], limited iron absorption inhibitors in traditional foods [53], infection increasing serum ferritin level [63], high levels of iron fortification in common foods [57], and high levels of iron supplementation [85].

### 4.4. Vitamin A

Of the 15 studies that examined the relationship between food insecurity and vitamin A [22,24,26,31,38,46,56,63,65,67,79,80,83,94,98], 7 found that food insecurity was significantly associated with lower serum vitamin A levels [24,26,31,56,79,83,94], and the remaining 8 studies found no significant association [22,38,46,63,65,67,80,98]. The studies which found lower vitamin A levels in populations with FI cited dietary deficiency, lack of sanitation, social and economic deprivation, and serious infections that deplete vitamin A stores as factors explaining the relationship [24,26,94]. Fourteen of the sixteen studies took place outside of the United States, namely, in South Asia, South America, and Africa. It is well-known that vitamin A deficiency clusters in regions of poverty, limited infrastructure, and high prevalence of infectious disease [109]. However, it is notable that nine studies found no significant associations. Reasons for lack of association include low prevalence of severe food insecurity due to sampling methods, small sample size, well-established food fortification programs, and robust hepatic maintenance of stable serum vitamin A levels [22,46,63,110].

### 4.5. Folate

Of the eight studies that examined the relationship between food insecurity and serum folate [31,33,45,57,67,79,84,89], three found that FI was significantly associated with lower serum folate levels [33,45,84], and the remaining five studies found no significant associations [31,45,57,67,79,89]. Studies that found lower folate levels cited severe food insecurity, poverty, decreased mobility, and decreased ability to care for oneself, particularly among the elderly, as factors contributing to the negative association [33,84]. In studies that found no significant associations between food insecurity and serum folate levels, government fortification programs and parents protecting children from food insecurity were cited as reasons for lack of association [33,67]. Notably, in Inuit populations, low-income individuals were more likely to consume the lower-cost, fortified food from government aid programs over the folate-poor traditional Inuit diet [111,112]. Hence, although these individuals were more likely to be food-insecure, their consumption of affordable fortified foods diminished the difference in folate deficiency between food-secure and food-insecure populations.

### 4.6. Vitamin D

None of the six studies that examined Vitamin D identified any association with food security [24,56,57,58,83,89]. Several of these studies were conducted in South Asia and the Middle East, where dressing habits, especially for women, cover the skin and prevent adequate absorption of Vitamin D [58,100]. Since these practices occur independently of food security status, they may mask differences between food-secure and -insecure populations.

### 4.7. Vitamin B12

Three studies found no association between vitamin B12 and FI [31,41,67], and two studies found a negative association [51,79]. Only one study provided an explanation for the observed lack of association that was specific to vitamin B12 [41]. The authors suggested that due to religious beliefs prohibiting nonvegetarian foods, widespread B12 deficiency may have minimized the magnitude of difference in B12 levels between food-insecure and food-secure individuals. No specific explanations were provided by the studies which found negative associations between FI and B12.

### 4.8. General

In addition to biomarker-specific reasons, there were several broadly-applicable explanations repeated across studies for why limited associations were found between biomarker levels and food security status. First, subgroup analysis identified 40 instances of pediatric studies finding no relationship between food insecurity and biomarkers assessed (supplemental Figure S4). In many of the pediatric studies, household food security status may not have reflected the actual food security status of the child whose biospecimen was collected. Multiple study authors remarked that intrahousehold food allocation practices may shield children from the effects of food insecurity [40,56,57,67,73,88].

Second, it is possible that in a broadly impoverished population, biomarker differences between individuals with and without FI are reduced [58,81]. Difficulties with access to foods rich in specific nutrients may create a broad background of nutrient insufficiency that masks differences in nutrient biomarkers [51]. Likewise, an unexpectedly high prevalence of certain health conditions in a population, such as metabolic syndrome, may affect serum levels of certain biomarkers and once again mask differences by reducing statistical contrast [96]. Covariates such as socioeconomic status, education level, or occupation also play roles in mediating participant health behaviors, but were not routinely adjusted for and thus may have reduced signal strength within studies.

Finally, biomarker measurements were taken at a single time point, but food insecurity was diagnosed from eating patterns lasting weeks to a year [31]. Thus, if participants consumed meals different from their usual eating pattern before sample collection, their observed biomarker levels may not actually correlate to typical values when on their standard diet. People with food insecurity may also have cyclical eating patterns, where food consumption may be increased during some points and lower at others [87]. This has been reported in studies in people receiving SNAP. Multiple biomarker measurements may be necessary to account for these fluctuations.

### 4.9. Strengths and Limitations

A particular strength of this review is that it utilized data from physiologic biomarker levels. Studies measuring levels based on intake are subject to bias from participant recall, inexact estimates of nutrient content, and inter-individual differences in nutrient absorption. Nevertheless, this review also has multiple limitations. Due to its exploratory nature, data quality was not systematically assessed. Furthermore, due to the considerable heterogeneity in study characteristics, the review did not pool data or include a weighted analysis by population size. As such, only the ultimate directionality of a biomarker’s association with food security status was tallied across studies. Inclusion criteria for acceptable food insecurity measurement tools were intentionally broad to allow for a comprehensive survey of the literature. Since the classification of participant food insecurity was carried out using a range of tools, the definition of food insecurity was not always consistent across studies.

Additionally, studies varied between measuring food security at the household versus individual level. Household food security was the more common measurement, but lacked the granularity to assess whether the participant whose biospecimen was being collected reflected the food security status of the household. This limitation may have been particularly relevant to studies in pediatric populations, since parents may protect their children from some of the effects of food insecurity, meaning that the household food insecurity status does not necessarily reflect the status of the child. Next, there are genetic components to certain deficiencies, and serum levels of nutrients are influenced by behaviors such as drinking and smoking and by conditions such as pregnancy. Serum levels of micronutrients may also be affected by supplementation, masking differences between populations. These variables were not always adjusted for between studies. Some of the included studies were conducted in highly specific populations with distinct dietary norms, which may limit the generalizability of conclusions to populations with different dietary practices. Finally, if only a subpopulation of a study sample showed a relationship between a biomarker and food security status, that study was still coded as demonstrating a relationship. This amplified the existence of potential relationships that may exist, but also reduced the data into a binary classification which may miss certain nuances.

### 4.10. Future Directions

As highlighted by the lack of consistent outcomes in existing studies, emphasis on single biomarkers to obtain an objective and quantitative assessment of dietary quality may be of limited value. Recent advances in nutritional untargeted metabolomics, when combined with traditional dietary assessments, may be an opportunity to develop shorter and more efficient nutrition status evaluation tools that combine abbreviated self-reported surveys with bioanalytic assessment [113,114,115]. As an adjunct to currently available dietary survey instruments, bioanalytic assessments can provide quantifiable estimates of dietary composition that are less prone to recall bias, reveal patterns of features that predict health outcomes, and identify metabolites associated with health outcomes that were not previously known (such as the recent example of identifying erythritol as a plasma biomarker associated with adverse cardiovascular events [116]). Such exploration may also capture nuances in health outcomes based on nutrient source—for example, it has been documented that for certain health outcomes, obtaining nutrients from supplements does not confer the same benefits as obtaining nutrients from food [117]. It should be noted though that many biomarkers are not specific to individual foods, so metabolomic quantification of exact dietary consumption is unlikely [14].

While bioanalytic methods offer increased objectivity, it is important to establish that they also face multiple limitations. First, pre-analytical handling has been shown to be a source of variation in metabolomic studies, with environmental exposure (e.g., light, oxidation), storage conditions (e.g., temperature, freeze–thaw cycles, preservatives), and timing of sample collection (e.g., circadian rhythm effects, effects of recent behaviors) all having been shown to affect reliability of result interpretation [118]. While generalizability of diet data captured from only one point in time is limited, it has nevertheless been demonstrated that multiple-sampling approaches, such as 24-h dietary recalls administered over three non-consecutive days, can approach the accuracy of more robust methods [119]. As such, it is plausible that repetitive biomarker measurements may provide more accurate estimates of habitual dietary patterns. Second, interindividual variation through genetic, environmental, and gut microbiota differences can confound findings. To overcome these effects, stratification of populations by “metabotype” may aid in signal detection [120]. Lastly, due to logistical constraints of large-scale sampling, metabolomic analyses may need to be conducted by various operators or laboratories, which generates batch effects that introduce artificial variation. While improved data-processing strategies help minimize these effects, they face compromises in statistical performance which may limit their utility [121].

Presently, food security measurement systems and policies focus more on food quantity than quality. Through exploration with bioanalytical methods, the dietary quality of FI individuals may be ascertained more accurately, and subcategories of FI that are reflective of nutrition security may be identified for more tailored intervention. Biomarker-based assessment methods will likely not supplant existing validated instruments for food security status assessment, but rather may serve as an adjunct to more holistically track outcome metrics of specific interventions and determine best strategies for combatting poor dietary quality in FI individuals.

## 5. Conclusions

Overall, the present review reveals limited scientific investigation into how biomarkers of dietary quality may differ between FI versus non-FI populations. Only three biomarkers of nutritional quality have been evaluated in this context. Of the 59 total biomarkers identified in this scoping review, few demonstrated consistent, directional relationships with food insecurity. Ultimately, future research is indicated to better characterize how nutrition security can be assessed in scalable, detailed formats for public health application.

## Data Availability

Not applicable.

## Supplementary Materials

The following supporting information can be downloaded at: https://www.mdpi.com/article/10.3390/nu15163576/s1; Figure S1: Search queries for each of the utilized databases; Figure S2: Biomarkers associated with food insecurity among all non-pregnant adults; Figure S3: Biomarkers associated with food insecurity among US non-pregnant adults; Figure S4: Biomarkers associated with food insecurity among all children; Figure S5: Biomarkers associated with food insecurity among US children; Figure S6: Biomarkers associated with food insecurity among all US non-pregnant adults and children; Figure S7: Biomarkers associated with food insecurity among studies using NHANES data.

## Appendix A

**Table A1:** Summary of characteristics for all studies (*n* = 78) included in the systematic review on nutritional biomarkers associated with food security status. SD = Standard Deviation, NR = not reported. (a) USDA = United States Department of Agriculture. (b) HDL = high-density lipoprotein. (c) USAID = United States Agency for International Development. (d) NHANES = National Health and Nutrition Examination Survey. (e) LDL = low-density lipoprotein. (f) PFAS = Per- and Polyfluorinated Substances. (g) PFNA = Perfluorononanoic acid. (h) PFOS = Perfluorooctane sulfonic acid. (i) PFOA = Perfluorooctanoic Acid. (j) PMNS = Pune Maternal Nutrition Study. (k) META-Health = Morehouse and Emory Team up to Eliminate Health Disparities. (l) DEHP = Di-2-ethylhexyl Phthalate. (m) DBP = Di-butyl-phthalate. (n) BzBP = benzylbutyl phthalate. (o) DEP = Di-ethyl-phthalate.

Author—Year	Country	Population Description	Population Size (N)	Population Age (SD)	% Female	Name of Food Security Assessment Tool	Source for Biomarker Assay	Fasting Status	Biomarker(s) Measured
Abdurahman—2021 [21]	Iran	Iranian adults with obesity	300	43.4 (10.9) years	84.1%	USDA ^a^ Household Food Security Status Questionnaire (modified for Iran)	Serum	Fasted	HDL ^b^; Triglycerides
Akelo—2014 [22]	Kenya	Pregnant Kenyan women attending their first antenatal care visit	505	24 years (median)	100%	USAID ^c^ Food and Nutrition Technical Assistance Project (FANTA) Household Food Insecurity Assessment Scale (HFIAS)	Serum	Nonfasted	Ferritin; Transferrin; Retinol-binding protein
Arango—2021 [23]	Colombia	Pregnant individuals	664	21 (7) years	100%	La Escala Latinoamericana y Caribeña de Seguridad Alimentaria (Latin American and Caribbean Food Security Scale (ELCSA))	Serum	Fasted	Ferritin
Baxter—2021 [24]	Pakistan	Unmarried and married, non-pregnant young women (15–23 years) living in rural Pakistan	3461	17.2 (1.2) years	100%	Household Food Insecurity Access Scale (HFIAS)	Serum	NR	Vitamin A; Ferritin; Vitamin D
Bayoumi—2020 [25]	Canada	Children aged 12–29 months	1245	18.1 months	47.8%	NutriSTEP questionnaire	Serum	NR	Ferritin
Carneiro—2020 [26]	Brazil	Representative sample of the population of children aged 6–59 months, treated at basic health units in the city of Rio de Janeiro, Brazil	519	6–23 months: 31% of population 24–59 months: 69% of population	49.8%	Brazilian Food Insecurity Scale	Serum	NR	Vitamin A
Chitekwe—2022 [27]	Nepal	Children aged 6–59 months	1709	NR	46.3%	Household Food Insecurity (HFI) Scale (included in the Nepal National Micronutrient Status Survey)	Serum	Fasted	Vitamin A
Corona—2021 [28]	United States	Predominately low-income, urban Black women	459	60.7 (12.93) years	81.7%	Custom: 90 min structured interview that included questions about food access	Blood (not specified)	NR	HDL; Total cholesterol
Crook—2021 [29]	United States	Adults aged 20 years or older participating in NHANES ^d^	7607	20–39 years: 37.5% of population 40–59 years: 40.1% of population ≥60 years: 22.4% of population	51.3%	Single question from USDA Household Food Security Survey Module	Serum	NR	Vitamin C
de Oliveira Campos—2016 [30]	Brazil	School children in Bahia, Brazil aged 6–14	1419	11 (2.83) years	48.7%	Brazilian scale of food insecurity	Urine	NR	Iodine
Dixon—2001 [31]	United States	Adults aged 20 years or older participating in NHANES III who were interviewed at home and examined at the mobile examination center	10,165	NR	52.1%	NHANES III Food Insufficiency Data	Serum	Fasted	Vitamin A; Vitamin B12; Vitamin B9; Vitamin C; HDL; LDL; ^e^ Triglycerides; Total cholesterol; Ferritin; Albumin, Alpha-carotene, Beta-carotene, Beta-cryptoxanthin, Lutein/zeaxanthin, Lycopene, Vitamin E
Dong—2021 [32]	United States	Urban American Indian/Alaska Native (AI/AN) youth	142	14 years	58.0%	Child Food Security Survey Module	Blood (not specified)	NR	Triglycerides; Total cholesterol
Duncan—2018 [33]	Canada	Non-pregnant women of reproductive age from Canadian Inuit communities	249	29.1 (6.0) years	100%	USDA Household Food Security Survey Module	Whole blood	Fasted	Vitamin B9
Egeland—2011 [34]	Canada	Canadian Inuit populations	2595	41 (14.7) years	62.0%	USDA Household Food Security Survey Module	Serum	Fasted	Ferritin
Egeland—2011 [35]	Canada	Indigenous Inuit preschoolers in 16 of the 25 communities of Nunavut	388	NR	NR	USDA Household Food Security Survey module, modified by Indian and Northern Affairs Canada for Inuit communities	Plasma	NR	Ferritin
Eicher-Miller—2009 [36]	United States	Nonpregnant children and adolescents from 3 to 19 years old who successfully completed the household interview and participated in the MEC examination	11,247	NR	45.1%	USDA Household Food Security Survey Module	Serum	NR	Ferritin
Eick—2022 [37]	United States	Pregnant individuals in the Chemicals In Our Bodies (CIOB) cohort	497	NR	100%	Household Food Security Scale (short form)	Serum	NR	PFAS ^f^ (PFNA ^g^, PFOS ^h^ acid, PFOA ^i^, methyl-perfluorooctane sulfonamide acetic acid (Me-PFOSA-AcOH), and perfluorohexanesulphonic acid (PFHxS)
Faramarzi—2019 [38]	Iran	Subjects who had participated in Azar cohort study	152	52.88 (9.08) years	NR	Household Food Insecurity Access Scale	Serum and whole blood	Fasted	Vitamin A; HDL; Triglycerides
Ford—2013 [39]	United States	Adults ≥ 20 years old participating in NHANES	10,455	43.8 years (median)	49.8%	USDA Household Food Security Survey Module	Whole blood	Fasted	HDL; Total cholesterol; urinary albumin-creatine ratio, non-HDL cholesterol
Fulay—2021 [40]	United States	Adolescents (12–17 years old) with household incomes at or below 300% federal poverty line	2876	NR	49.1%	USDA Household Food Security Survey Module	Whole blood	Fasted	HDL; LDL; Triglycerides; Total cholesterol
Ganpule-Rao—2020 [41]	India	18-year-old children living in one of six rural villages near Pune, Maharashtra, India, born to mothers studied in the larger PMNS^j^ study	418	18 years	46.7%	Number and type of food shops/1000 population, water availability, and distance from the highway	Plasma	Fasted	Vitamin B12
Gebreegziabher—2017 [42]	Ethiopia	Non-pregnant women aged 18 and older in rural communities of Sidama, southern Ethiopia	202	30.8 (7.8) years	100%	Household Food Security Access Scale (HFIAS)	Plasma and whole blood	Fasted	Ferritin; Transferrin; Plasma iron, Soluble transferrin receptors
Gebreegziabher—2022 [43]	Ethiopia	School-aged children	408	9 (1.8) years	50.5%	Household Food Insecurity Access Scale (HFIAS) and Household Hunger Scale (HHS)	Urine	NR	Aflatoxin M1
Gebremedhin—2011 [44]	Ethiopia	Pregnant women	700	28.5 (5.5) years	100%	Household Food Insecurity Access Scale (HFIAS)	Serum	Both fasted and nonfasted	Zinc
Gowda—2012 [45]	United States	Adults ≥ 18 years old participating in NHANES	12,191	NR	NR	USDA Household Food Insecurity Survey	Whole blood	Fasted	Vitamin B9
Gubert—2016 [46]	Brazil	Brazilian children under 5 years old	4064	NR	NR	Brazilian Household Food Insecurity Measurement Scale	Whole blood	NR	Vitamin A
Habib—2016 [47]	Pakistan	Pakistani children aged 6–59 months	7138	NR	48.3%	Household food insecurity access scale (HFIAS) developed by Food and Nutrition Technical Assistance (FANTA) project	Serum	NR	Vitamin A; Ferritin; Zinc
Habib—2018 [48]	Pakistan	Non-pregnant Pakistani women	7491	NR	100%	Household food insecurity access scale (HFIAS) developed by Food and Nutrition Technical Assistance (FANTA) project	Serum	NR	Vitamin A; Ferritin; Zinc
Hamedi-Shahraki—2021 [49]	Iran	Women of reproductive age at health centers affiliated with Zabol University of Medical Sciences	630	33.2 (7.8) years	100%	Household Food Insecurity Access Scale (HFIAS) questionnaire validated in an Iranian population	Serum	Fasted	HDL; LDL; Triglycerides; Total cholesterol
Hanson—2018 [50]	United States	Pregnant women recruited from the Labor and Delivery unit in a Midwestern United States Academic Medical Center at the time of delivery	180	28.7 (5.6) years	100%	US Household Food Security Survey Module (US HFSSM); USDA Economic Research Service national food desert database at the census-tract level	Serum	Nonfasted	Vitamin A; Vitamin E; Beta-cryptoxanthin; Lutein/zeaxanthin; Beta-carotene; Lycopene
Herran—2015 [51]	Colombia	Children and women (pregnant and non-pregnant)	9024	NR	50.4% of kids, 100% of pregnant women	Community Childhood Hunger Identification Project (modified version)	Serum	NR	Vitamin B12
Holben—2006 [52]	United States	Adults ≥ 18 years old from 6 rural counties in Ohio	2580	44.7 (18) years	66.1%	USDA Household Food Security Survey Module	Whole blood	Nonfasted	Total cholesterol
Jamieson—2011 [53]	Canada	Non-pregnant Inuk women ≥ 18 years old from Arctic Canada	697	42 (15) years	100%	USDA Household Food Security Survey Module	Serum	Fasted	Ferritin
Jamieson—2012 [54]	Canada	Inuit men	880	42 (15) years	0.0%	USDA Household Food Security Survey Module	Whole blood	Fasted	Ferritin
Jamieson—2013 [55]	Canada	Non-pregnant, self-identified Inuk women	1550	42 (15) years	100%	USDA Household Food Security Survey Module	Serum	Fasted	Ferritin
Janmohamed—2020 [56]	Mongolia	Mongolian children aged 6–23 months	938	NR	50.9%	Household Food Insecurity Access Scale	Serum	NR	Vitamin A; Ferritin; Vitamin D
Jun—2021 [57]	United States	US children aged 1–18 years	9147	NR	49.4%	USDA Household Food Security Survey Module	Whole Blood	Fasted	Vitamin B9; Ferritin; Zinc; Vitamin D
Kazemi—2020 [58]	Azerbaijan	Women of reproductive age	266	40.93 (11.1) years	100%	Household Food Security Scale (short form)	Serum	Fasted	Ferritin; Vitamin D
Kelli—2017 [59]	United States	Adults aged 20 to 79 years and residing in the Atlanta metropolitan area participating in the META-Health ^k^ study or the Predictive Health study	1421	49.4 (10.2) years	61.5%	USDA Food Access Research Atlas	Serum	Fasted	HDL; LDL; Triglycerides; Total cholesterol
Kim—2020 [60]	United States	Adults 30–70 years old who self-identified as Black or African American from local communities of the Atlanta-Sandy Springs-Alpharetta, Georgia, metropolitan area	394	52.8 (10.3) years	61.0%	Neighborhood Health Questionnaire	Plasma	Fasted	HDL; LDL; Triglycerides; Total cholesterol
Kobrosly—2012 [61]	United States	Women aged 20–39 years participating in NHANES	1182	29 years (median)	100%	USDA Household Food Security Survey Module	Urine	Adjusted for fasting time	Phthalates: four metabolites of DEHP:^l^ mono-2-ethylhexyl phthalate (MEHP), mono-(2-ethyl-5-oxohexyl) phthalate (MEOHP), mono-(2-ethyl-5-hydroxyhexyl) phthalate (MEHHP), and mono-(2-ethyl-5-carboxypentyl) phthalate (MECPP); two metabolites of DBP: ^m^ mono-n-butyl phthalate (MnBP), and monoisobutyl phthalate (MiBP); one metabolite of BzBP: ^n^ mono-benzyl phthalate (MBzP); one metabolite of DEP: ^o^ mono-ethyl phthalate (MEP)
Landry—2019 [62]	United States	Hispanic/Latino children in elementary schools that offered the after-school program “LA’s Best”	218	Food-secure population: 9.3 (0.9) years Food-insecure population: 8.9 (0.8) years	50.9%	Child Food Security Assessment (CFSA)	Blood (not specified)	Fasted	HDL; LDL; Triglycerides; Total cholesterol
Leyna—2010 [63]	Tanzania	Adults in Oria Village	1014	NR	69.1%	Radimer/Cornell food insecurity measure (validated in rural Kilimanjaro, Tanzania)	Plasma	NR	Vitamin A; Ferritin
Li—2017 [64]	Colombia	Colombian children	14,559 had ferritin levels analyzed, 4279 had zinc levels analyzed, 3844 had vitamin A levels analyzed	NR	61.1% (ferritin)	Community Childhood Hunger Identification Project (adapted for and validated in a Colombian population)	Plasma and serum	NR	Vitamin A; Ferritin; Zinc
Mahfuz—2019 [65]	Bangladesh	Children with low socioeconomic status and limited sanitary conditions in a slum settlement in Bauniabadh in the Mirpur area of Dhaka city	153 had ferritin levels analyzed, 154 had zinc levels analyzed, 155 had retinol levels analyzed	7 months	52.4%	Household Food Insecurity Access Scale (HFIAS) developed by the Food and Nutrition Technical Assistance (FANTA) project	Plasma	NR	Vitamin A; Ferritin; Zinc
Maldonado—2022 [66]	United States	Hispanic/Latino youth aged 8 to 16 years	1325	High food security population: 12.2 (2.6) years Marginal food security population: 11.7 (2.7) years Low food security population: 12.4 (2.5) years Very low food security population: 12.3 (2.6) years	50%	USDA Household Food Security Survey Module	Serum	NR	HDL; Triglycerides
Marín—2021 [67]	Colombia	Bogotá school cohort formed in February 2006 by randomly recruiting 3202 children from 5 to 12 years who studied in the public schools of Bogotá and belonged to the middle and low socioeconomic strata	2660	8.7 (1.8) years	49.6%	Household Food Security Survey Module (HFSSM) validated in Spanish	Serum	NR	Vitamin A; Vitamin B12; Vitamin B9; Ferritin; Zinc
Mazidi—2018 [68]	United States	Adults ≥ 18 years old	3876	48.1 years	51.4%	USDA Household Food Security Survey Module	Plasma	NR	Trans-9-hexadecenoic acid; Trans-11-octadecenoic acid; trans-9-octadecenoic acid; trans-9-, trans-12-octadecenoic acid
Mohajeri—2022 [69]	Iran	Adults aged 35–65 years selected by random sampling from the participants of the Persian Cohort Study	500	45 years	62.4%	USDA Household Food Security Survey Module	Serum	Fasted	LDL; Triglycerides; Total cholesterol
Mohamadpour—2012 [70]	Malaysia	Indian women working on palm plantations	147	NR	100%	Radimer/Cornell Hunger and Food Insecurity Instrument	Serum	Fasted	HDL; LDL; Total cholesterol
Murillo-Castillo—2018 [71]	Mexico	Mothers of children attending elementary school (grades 1–5) in Kino Bay, a fishing community located in Northwest Mexico	116	36.4 (8.9) years	100%	Mexican Scale of Food Security	Serum	Fasted	HDL; Total cholesterol
Nguyen—2014 [72]	Vietnam	Women of reproductive age living in the mountainous region of Northeast Vietnam	4986	26.2 (4.6) years	100%	Household Food Insecurity Access Scale	Serum	NR	Ferritin
Nikniaz—2018 [73]	Iran	Individuals from districts of East Azerbaijan	253	Men: 43.56 (14.15) years Women: 41.14 (11.16) years	55.7%	Household Food Security Scale (short form)	Serum	Fasted	HDL; LDL; Triglycerides; Total cholesterol
Nur Atiqah—2015 [74]	Malaysia	Students from all departments of Universiti Teknologi MARA Puncak Alam	124	NR	87.9%	US Adult Food Security Survey Module	Serum	Fasted	HDL; LDL; Triglycerides; Total cholesterol
Pak—2021 [75]	United States	Individuals aged 50–100 years in the 2006–2014 Health and Retirement Study cohort	14,394	60 (IQR 56–69) years	56.6%	Two Item Household Food Insecurity Screener	Whole blood	NR	HDL; Total cholesterol
Park—2014 [76]	United States	Pregnant women	1045	NR	100%	USDA Food Security Survey Module	Serum	NR	Ferritin; Transferrin
Park—2020 [77]	United States	Female adults	4249	NR	100%	USDA Household Food Security Scale	Serum	Fasted	HDL; Triglycerides
Pasricha—2010 [78]	India	Children aged 12 to 23 months in 2 rural districts of Karnataka, India	401	17.2 months (IQR 16.8–17.5)	49.7%	Household Food Insecurity Access Scale	Serum	NR	Vitamin A; Vitamin B12; Vitamin B9; Ferritin
Pasricha—2011 [79]	India	Children 12–23 months old in rural India	405	17.2 months (IQR 16.8–17.5)	49.6%	Household Food Insecurity Access Scale	Serum	NR	Ferritin
Pedraza—2014 [80]	Brazil	Preschool children attending public children’s gardens of the government of the State of Paraíba, Brazil	193	NR	NR	Brazilian Food Insecurity Scale	Serum	NR	Vitamin A; Zinc
Pinzón-Rondón—2019 [81]	Colombia	Columbian children 12–59 months	4275	2.66 (1.09) years	46.8%	Latin-American and Caribbean household food security scale (ECLA)	Serum	Nonfasted	Zinc
Pirkle—2014 [82]	Canada	Inuit children from Nunavik whose mothers participated in either the Cord Blood Monitoring Program or Environmental Contaminants and Infant Development Study	294	11.3 (0.8) years	50.3%	USDA Household Food Security Survey Module	Serum	NR	Ferritin
Pobee—2020 [83]	Ghana	Ghanian women planning to become pregnant	95	NR	100%	USDA Household Food Security Survey Module	Serum and plasma	NR	Vitamin A; Ferritin; Transferrin; Zinc; Vitamin D; Copper
Sahyoun—2000 [84]	United States	Adults aged 65 years and older participating in NHANES	3855	72.3 years	52.0%	Custom: One-question about food sufficiency	Serum	NR	Vitamin B9; Vitamin C; Lutein/zeaxanthin; beta-cryptoxanthin; beta-carotene; Vitamin E
Salarkia—2015 [85]	Iran	Children of Iranian mother-child pairs	423	15.1 (5.7) months	46.1%	Household Food Insecurity Access Scale	Serum	Nonfasted	Ferritin
Sattler—2016 [86]	United States	Adults aged 20 and older participating in NHANES	7439	44.12 (12.97) years	48.0%	USDA Household Food Security Survey Module	Serum	NR	Total cholesterol
Shariff—2014 [87]	Malaysia	Malay and Indian, non-pregnant, non-lactating women aged 19–49 years from rural and urban low-income households	625	38 years	100%	Radimer/Cornell Hunger and Food Insecurity Instrument	Serum	Fasted	HDL; LDL; Triglycerides; Total cholesterol
Shin—2015 [88]	United States	Healthy adults from the 2008–2011 Survey of the Health of Wisconsin	1663	NR	48.0%	Custom: Two-question food insecurity screener	Serum	Nonfasted	HDL; Total cholesterol
Shiue—2016 [89]	United States	Adults ≥ 20 years old	4979	NR	52.1%	USDA Household Food Security Survey Module	Serum, urine	Fasted	Vitamin B9; Vitamin C; HDL; Total cholesterol; Phthalates, Vitamin D, apolipoprotein, heavy metals (barium, beryllium, cadmium, cobalt, cesium, molybdenum, lead, platinum, antimony, thallium, tungsten, uranium), bisphenols (4-tert-octylphenol, benzophenone-3, bisphenol A, triclosan), pesticides (2,5-dichlorophenol, O-phenyl phenol, 2,4-dichlorophenol, 2,4,5-trichlorophenol, 2,4,6-trichlorophenol)
Smalls—2020 [90]	United States	Grandparents residing in Appalachia, Kentucky, who were the primary caretakers for their grandchildren	65	59.4 (7.4) years	98.5%	USDA Household Food Security Survey Module	Whole blood	Fasted	HDL; LDL; Triglycerides; Total cholesterol
Suarez—2015 [91]	United States	Adults aged 20 and older participating in NHANES	22,173	46.7 (14.5) years	51.9%	Food Desert US Census Tract Data	Serum	NR	Alpha-carotene; beta-carotene; beta-cryptoxanthin; Lutein/zeaxanthin
Tayie—2009 [92]	United States	Adults aged 18–50 participating in NHANES	5549	NR	50.3%	USDA Adult Food Security Survey Module	Serum	Fasted	HDL; LDL; Triglycerides; Total cholesterol
Tayie—2019 [93]	United States	Adults aged 18–65 participating in NHANES	2780	41.6 (0.4) years	51.5%	USDA Household Food Security Survey Module	Serum	NR	Copper
Tester—2016 [94]	United States	Low-income adolescents	1072	Food-secure population: 15 (14.9–15.1) Marginal food security population: 14.7 (14.5–14.9) Low food security population: 14.3 (14.1–14.5) Very low food security population: 14.8 (14.6–15.0)	47.9%	USDA Household Food Security Survey Module	Serum	Fasted	HDL; LDL; Triglycerides; Total cholesterol
van Woerden—2019 [95]	United States	Adults aged 18 and older participating in NHANES	9886	NR	50.0%	USDA Adult or Household Food Security Survey Module	Urine	NR	Bisphenol A
Weigel—2007 [96]	United States	Migrant and seasonal farmworkers	100	NR	43.0%	USDA Household Food Security Survey Module	Whole blood	Fasted	HDL; Triglycerides; Total cholesterol
Weigel—2016 [97]	Ecuador	Adult female heads of households and their minor children aged 6–12 years	794	34 (10.6)	100%	Quito Household Food Security Survey Module (adapted from USDA HFSSM)	Whole blood	Fasted	HDL; LDL; Triglycerides; Total cholesterol
Yeudall—2007 [98]	Uganda	Children 2 to 5 years old	296	NR	NR	Custom: novel questionnaire developed from prior research in this region	Whole blood	Nonfasted	Vitamin A
