# Supplementary material for: A Scoping Review of Nutritional Biomarkers Associated with Food Security
Source: Nutrients. 2023 Aug 14;15(16):3576. doi: 10.3390/nu15163576 (PMC10459650; doi:10.3390/nu15163576)
Supplement: Supplementary file 1 [file nutrients-15-03576-s001.zip › nutrients-2539746-supplementary Figure S1.pdf]

Supplemental Figure 1: Search queries for each of the utilized databases. MeSH = Medical Subject Heading.

**Database**	**Search Query**
PubMed	("food secur*"[tw] OR “food security”[MeSH] OR "food insecur*"[tw] OR “food insecurity”[MeSH] OR "food access"[tw] OR "food desert*"[tw] OR “food deserts”[MeSH] OR "food swamp*"[tw] OR "food sufficien*"[tw] OR "food insufficien*"[tw]) AND (metabolite*[tw] OR metabolom*[tw] OR “metabolome”[MeSH] OR “metabolomics”[MeSH] OR "metabolic syndrome*" OR “nutrition surveys”[MeSH] OR “nutrition survey*”[tw] OR "diet survey*"[tw] OR "diet questionnaire*"[tw] OR "diet screener*"[tw] OR "dietary nutrient*"[tw] OR blood[tw] OR blood[MeSH] OR blood[subheading] OR urine[tw] OR urine[MeSH] OR urine[subheading] OR plasma[tw] OR hair*[tw] OR nail*[tw])
Scopus	TITLE-ABS-KEY (("food secur*" OR "food insecur*" OR "food access" OR "food desert*" OR "food swamp*" OR "food sufficien*" OR "food isufficien*" AND ("metabolite*" OR "metabolom*" OR "metabolic syndrome*" OR "diet survey*" OR "diet questionnaire*" OR "diet screener*" OR "dietary nutrient*" OR blood OR urine OR plasma OR hair* OR nail*))
AGRICOLA	("food secur*" OR "food insecur*" OR "food sufficien*" OR "food insufficien*" OR "food access" OR "food desert*" OR "food swamp*") AND (metabolite* OR metabolom* OR "metabolic syndrome*" OR "diet survey*"

	OR "diet questionnaire*" OR "diet screener*" OR "dietary nutrient*" OR blood OR urine OR plasma OR hair* OR nail*)
Embase	('food secur*':ti,ab,kw OR 'food security'/exp OR 'food insecur*':ti,ab,kw OR 'food insecurity'/exp OR 'food access':ti,ab,kw OR 'food desert':ti,ab,kw OR 'food swamp*':ti,ab,kw OR 'food sufficien*':ti,ab,kw OR 'food insufficien*':ti,ab,kw) AND ('metabolite*':ti,ab,kw OR 'metabolite'/exp OR 'metabolom*':ti,ab,kw OR 'metabolomics'/exp OR 'metabolome'/exp OR 'metabolic syndrome*':ti,ab,kw OR 'metabolic syndrome x'/exp OR 'diet survey*':ti,ab,kw OR 'diet questionnaire*':ti,ab,kw OR 'diet screener*':ti,ab,kw OR 'nutrition survey*':ti,ab,kw OR 'dietary nutrient*':ti,ab,kw OR 'blood':ti,ab,kw OR 'blood'/exp OR 'plasma':ti,ab,kw OR 'urine':ti,ab,kw OR 'urine'/exp OR 'hair'/exp OR 'hair*':ti,ab,kw OR 'nail'/exp OR 'nail*':ti,ab,kw) NOT (('food secur*':ti,ab,kw OR 'food security'/exp OR 'food insecur*':ti,ab,kw OR 'food insecurity'/exp OR 'food access':ti,ab,kw OR 'food desert':ti,ab,kw OR 'food swamp*':ti,ab,kw OR 'food sufficien*':ti,ab,kw OR 'food insufficien*':ti,ab,kw) AND ('metabolite*':ti,ab,kw OR 'metabolite'/exp OR 'metabolom*':ti,ab,kw OR 'metabolomics'/exp OR 'metabolome'/exp OR 'metabolic syndrome*':ti,ab,kw OR 'metabolic syndrome x'/exp OR 'diet survey*':ti,ab,kw OR 'diet questionnaire*':ti,ab,kw OR 'diet screener*':ti,ab,kw OR 'nutrition survey*':ti,ab,kw OR 'dietary nutrient*':ti,ab,kw OR 'blood':ti,ab,kw OR 'blood'/exp OR 'plasma':ti,ab,kw OR 'urine':ti,ab,kw OR 'urine'/exp))
