# Supplementary material for: A Scoping Review of Nutritional Biomarkers Associated with Food Security
Source: Nutrients. 2023 Aug 14;15(16):3576. doi: 10.3390/nu15163576 (PMC10459650; doi:10.3390/nu15163576)
Supplement: Supplementary file 1 [file nutrients-15-03576-s001.zip › nutrients-2539746-supplementary Figure S7.pdf]

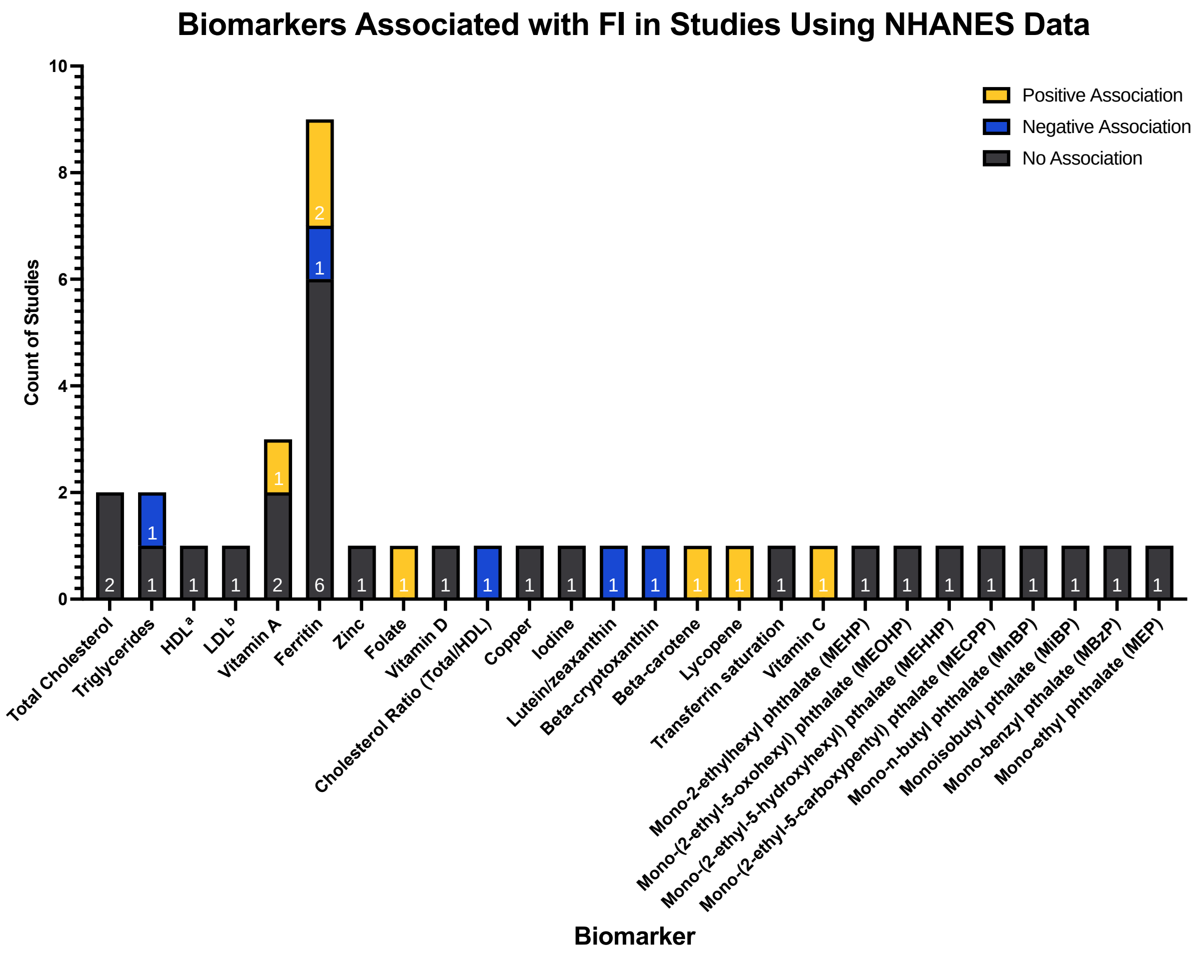

Supplemental Figure 7: Biomarkers associated with food insecurity among studies using NHANES data.

Key:

[a] = High density lipoprotein

[b] = Low density lipoprotein
